# The Effect of Cooking Method and Cooked Color on Consumer Acceptability of Boneless Pork Chops

**DOI:** 10.3390/foods11010106

**Published:** 2021-12-31

**Authors:** Lauren T. Honegger, Erin E. Bryan, Hannah E. Price, Taylor K. Ruth, Dustin D. Boler, Anna C. Dilger

**Affiliations:** 1 Department of Animal Sciences, University of Illinois, 1503 S. Maryland Drive, Urbana-Champaign, IL 61801, USA; honeggr2@illinois.edu (L.T.H.); eebryan2@illinois.edu (E.E.B.); hprice3@illinois.edu (H.E.P.); dboler2@illinois.edu (D.D.B.); 2 Department of Agriculture Leadership, Education and Communication, University of Nebraska, 143 Finley Hall, P.O. Box 830947, Lincoln, NE 68583, USA; taylor.ruth@unl.edu

**Keywords:** consumer preference, cooked color, degree of doneness, grill, pork, sous-vide

## Abstract

The objective was to determine the effects of sous-vide cooking and degree of doneness on consumer eating experience of pork chops when cooked color was expected to differ. The hypothesis was consumers would prefer a cooked brown color and would rate grilled chops more acceptable than sous-vide chops. Chops were cooked to 63 °C or 71 °C using either an open-hearth grill or a sous-vide device. Participants evaluated four samples for tenderness, juiciness, flavor, and overall acceptability. Participants rated a greater percentage of chops cooked sous-vide at 63 °C as tender (82.82%), juicy (55.83%) and acceptable (60.34%) compared with all other cooking method and degree of doneness combinations. Participants rated a greater percentage of sous-vide chops as tender and acceptable compared to grilled chops. Participants rated a greater percentage of chops cooked to 63 °C as tender, juicy, flavorful, and acceptable when compared to 71 °C. Even when participants could visualize cooked color, they preferred chops cooked to 63 °C compared with chops cooked to 71 °C. Overall, participants preferred chops cooked to 63 °C compared to 71 °C regardless of the cooking method and preferred chops cooked to 63 °C using the sous-vide cooking method the most among all treatments.

## 1. Introduction

Sensory tenderness scores improve when pork is cooked to a lesser endpoint cooking temperature [1,2,3]. As evidence, sensory tenderness scores improved as internal cooking temperature decreased from 80 °C to 62 °C [2], and 48% of consumers rated pork chops cooked to 63 °C as acceptable compared to only 14% of consumers when chops were cooked to 71 °C [3]. Due to the improvement in sensory traits without compromising food safety, in 2011 the United States Department of Agriculture (USDA) revised the recommended final internal cooking temperature of whole pork muscles from 71 °C to 63 °C [4]. As with any change in guidelines, consumers are often unaware or unwilling to alter their cooking methods. Most consumers tend to use the “eye ball” test to determine doneness rather than a meat thermometer to determine degree of doneness or they cook pork until no red or pink is present in the center [5]. It is possible that consumers have predetermined biases about the safety of pork and therefore preferences for the internal color appearance of that may skew their judgments of acceptability when allowed to view cooked color. In previous work, consumers were presented chops prepared using an immersion heater sous-vide cooking device under red lighting to mask cooked color differences between different degrees of doneness [3]. It is well-documented that as internal temperature increases, pork will appear less pink internally [6]. However, there are limited data on the effect degree of doneness on consumer acceptability of pork chops when consumers are given the chance to visually appraise the chop and then evaluate sensory characteristics.

Further, sous-vide cooking is becoming increasingly more popular among consumers [1,7]. An advantage of sous-vide cooking is the product can be cooked longer without heating the meat to greater than the desired internal temperature set by the immersion cooker [7]. However, using a sous-vide cooking method does not allow Maillard reaction to occur, which causes browning on the surface of the meat and development of cooked flavor [8]. This raises the question whether consumers would find meat cooked in a sous-vide style without searing, and therefore lacking in browning, acceptable. Psychological factors such as risk (e.g., food safety) greatly influence consumers’ ultimate eating experience [9]. The hypothesis was that the reduction in internal browning due to reduced cooking temperatures and lack of external browning from sous-vide cooking would result in consumers finding pork chops cooked to 63 °C using sous-vide less acceptable due to predetermine biases. This would result in less overall acceptability scores compared with other cooking temperature and cooking method combinations.

The objective was to test this hypothesis and determine the effect of cooking method and degree of doneness on consumer eating experience of pork chops when consumers were able to observe differences in cooked color. An additional aim of this study was to determine the habits and attitudes of consumers regarding their behavior around cooking pork, especially regarding degree of doneness.

## 2. Materials and Methods

Pigs from a single source were slaughtered at a commercial facility under the supervision of the USDA Food Safety and Inspection Service. Boneless loins were purchased from the facility and transported to the University of Illinois Meat Science Laboratory. Therefore, no Institutional Animal Care and Use Committee approval was needed. Sensory procedures for consumer evaluations were reviewed and accepted by the University of Illinois Office for the Protection of Research Subjects prior to recruitment.

### 2.1. Loin Origin

Visual color (6-point visual scale) [10] and marbling (10-point visual scale) [10] were evaluated, and loins (12 total) were visually selected at the abattoir to represent an average pork loin for visual color (score 2–3) and visual marbling (score 2–3) of U.S. retail pork chops [11] and then vacuumed-sealed. Boneless loins were transported to the University of Illinois Meat Science Laboratory. Upon arrival, loins were aged at 4 °C until 10 days (d) postmortem, then frozen as whole boneless loins at −20 °C for less than 6 months. Frozen loins were cut into 3.2 cm thick chops using a Biro Meat Saw (model 3334, The Biro MGF. Co., Marblehead, OH, USA) beginning at the anterior end of the loin. Chops containing the spinalis dorsi muscle were excluded. The first 9 chops posterior to the spinalis dorsi muscle were cut and saved from each loin. Frozen chops were vacuum packaged, placed into boxes according to assigned panel, and stored at −20 °C until sensory evaluations. Twenty-four hours prior to each panel, the assigned panel box was removed from the freezer and chops were thawed at 4 °C. Chop 1 (chop at approximately the area of the 10th rib) was used for quality measurements and then discarded after evaluation. Chop number 2, 3, 4, and 5 were randomly assigned one of four cooking method and degree of doneness combinations: grill, 63 °C; sous-vide, 63 °C; grill, 71 °C; sous-vide, 71 °C. The same procedure was followed for chops 6, 7, 8, and 9. This resulted in one chop from each loin for quality measurements and a total of 96 chops (8 from each loin) for sensory evaluations.

### 2.2. Chop Quality Measurements

Chop 1 from each loin (*n* = 12) was used to measure ultimate pH, visual appraisals, and instrumental color and used represent each loin (Table 1). Ultimate pH was measured with a REED SD-230 meter (Wilmington, NC, USA) fitted with a FC 200 B series electrode (Hanna Instruments; Woonsocket, RI, USA). Visual color (6-point visual scale) [10], marbling (10-point visual scale) [10], and subjective firmness (5- point subjective scale) [12] were measured on the chop face by a trained technician. Instrumental color (L*, a*, and b*) [13] was measured with a Konica Minolta CR-400 colorimeter (Minolta Camera Company, Osaka, Japan) using D65 illuminant, 2° observer angle, and an 8-mm closed aperture.

### 2.3. Consumer Sensory Panels

Consumer sensory panels were conducted at the University of Illinois Meat Science Laboratory. To assemble a pool of consumers for these panels, email lists and flyers were used for recruitment. Consumers were asked to fill out an online survey regarding their demographic information [age, gender, race, and education level (Table 2)]. Additionally, consumers recorded their availability for scheduled evaluation days. Each of 3 evaluation days had a total of 8 panels lasting a total of 30 min per panel. Each panel could accommodate up to 8 consumers at a time. Each consumer panel used 4 total chops all originating from the same loin to fill a 2 × 2 factorial arrangement of cooking method (sous-vide or grill) and degree of doneness (63 °C or 71 °C) combinations.

Four hours prior to the first panel of the day, immersion heater sous-vide devices (ANOVA Precision Cooker, Anova Applied Electronics, San Francisco, CA, USA) were placed in water baths and water was heated to the correct cooking temperature (63 °C or 71 °C). Vacuum sealed chops were placed into water baths 2 h prior to each panel. Temperatures for the warm water baths were monitored during the cooking process. Two additional chops from each loin were cooked on a Farberware Open Hearth grill (model 455 N, Walter Kidde, Bronx, NY, USA). Chops were monitored using a copper-constantan thermocouple (Type T, Omega Engineering, Stamford, CT, USA) connected to a digital scanning thermometer (model 92000-00, Barnat Co., Barrington, IL, USA). Thermocouples were placed in the geometric center of the chop and placed on the grill. Chops were flipped at an internal temperature of 31.5 °C or 35.5 °C, then remained on the grill until chops reach either 63 °C or 71 °C. Chops were removed from the grill or water bath and final internal temperatures were measured using a meat thermometer.

After cooking, chops were sliced perpendicular to the length of the chop to expose the center of the chop. Internal cooked color was measured using a Minolta CR-400 Chroma meter (Minolta Camera Co., Ltd., Osaka, Japan) using a D65 light source, 2° observer angle, an 8 mm aperture, and calibrated using a white tile. Instrumental color readings included: lightness (L*), redness (a*), and yellowness (b*) [13]. Then, chops were placed in a sample sizer and sliced into 1 cm × 1 cm × 3.2 cm samples. Two pieces from each chop were placed in a small plastic cup with a numbered lid before being served to consumers. Chops from all 4 combinations were served hot immediately after cooked color evaluations.

**Figure 1:**
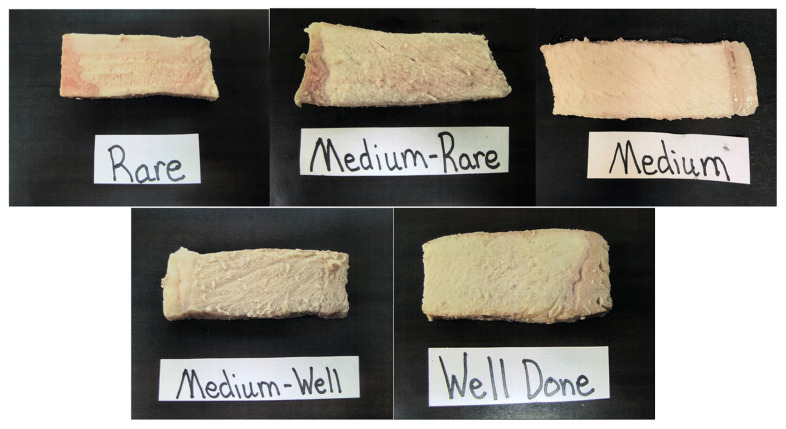
Set of photographs displaying chops cooked to various degrees of doneness used for pre- and post-survey questions. Images display a cooked chop illustrating rare, medium-rare, medium, medium-well, and well done.

Upon arrival, participants were provided a packet of instructions. First, informed consent forms were signed before the panel session could begin. Once all participants were present and signed consent, a pre-survey was given with 7 questions. Question 1 asked participants how often they consumed pork and question 2 asked participants how often they prepared pork from the following options: more than once a week, once a week, 2–3 times a month, once a month, or less than once a month. Question 3 asked participants to select all the methods they use to cook pork from the following options: stove top, oven, grill, air fryer, deep fryer, slow cooker, sous-vide, or other. Question 4 asked participants to select all the methods they use to determine when pork is done and safe to consume at home from the following options: use a meat thermometer, look at the color of the meat, cook until juices run clear, cook for a specific amount of time, I do not check to see if it is done, or other. Questions 5 asked participants to evaluate a set of five photos, reviewed by meat science specialists, displaying chops cooked to various degrees of doneness that ranged from rare to well done (Figure 1). Participants were asked to select the photo that represented their preferred degree of doneness from the photos, options included: rare, medium rare, medium, medium well, and well done. Question 6 asked participants to select the main reason why they chose the degree of doneness preference from the previous question from the following options: best flavor, best texture, juiciest, safest to consume, and other. The final question asked participants to circle what temperature they believed pork is safe to consume, temperatures ranged from 100 °F (37.7°C) to 200 °F (93.3 °C) and increased in 5 °F (0.56 °C) increments. 

After completion of the pre-survey, a brief set of instructions were provided to participants regarding the evaluation sheet and what to expect during the sensory panel. Participants were seated in a breadbox style sensory booth room under white florescent light to allow participants to observe differences cooked color between chops. In each sensory booth, participants were provided unsalted crackers and water to use as palette cleanser between samples. Upon eating a sample, participants were asked to rate it for tenderness, juiciness, flavor, and overall acceptability. A nine-point scoring system was used for each of the previously described quality attributes, where 1 was extremely tough, extremely dry, extremely bland, and unacceptable, 5 was neutral for tenderness, juiciness, flavorful, and acceptability, and 9 was extremely tender, extremely juicy, extremely flavorful, and acceptable. Each participant within a panel was served 4 samples, representing all treatment combinations (grill, 63 °C; sous-vide, 63 °C; grill, 71 °C; sous-vide, 71 °C), in a randomized order.

Once all samples were served and evaluated by participants, participants were asked to return their evaluation sheets to the moderator. The moderator then revealed cooking methodology and degree of doneness for each sample to the participants. Once all the sample identities were revealed, the moderator asked participants to discuss how they ranked each sample and their thoughts with how the chops were prepared. Participants were asked to fill out a post-test survey to determine if views and beliefs of participants changed over the course of the session. Post-test questions included some of the same questions from the pre-test survey. Participants were again asked to evaluate the set of 5 photos and pick their preferred degree of doneness (Figure 1), select the main reason for why they chose that degree of doneness, and to circle what temperature they believed pork is safe to consume.

### 2.4. Statistical Analysis

Summary statistics for loin quality measurements were calculated using the MEANS procedure of SAS (SAS Inst. Inc., Cary, NC, USA). Cooked color data were analyzed using the MIXED procedure of SAS using cooking method, degree of doneness, and the interaction between cooking method and degree of doneness in the model. All means were separated using the PDIFF option and were considered significantly different from 0 at *p* < 0.05. Panelist scores for each sample for tenderness, juiciness, flavor, and overall acceptability were entered into Excel (Microsoft Corporation, Redmond, WA, USA, 2016). The scoring was sorted into three categories. Scores 1 through 3 were considered not tender, not juicy, not flavorful, or unacceptable. Scores 4 through 6 were considered neutral for tenderness, juiciness, flavor, and overall acceptability. Scores 7 through 9 were considered tender, juicy, flavorful, and acceptable [3]. Data were analyzed in the exact manner previously reported [3] with the GLIMMIX procedure of SAS using cooking method, degree of doneness, and the interaction as fixed effects and panel as a random variable. Means were separated using the PDIFF option and were considered significant at *p* < 0.05. These means represented the percentage of panelists who were represented in each of the three scoring categories.

Survey results were divided into pre- and post-survey questions. Pre- and post-survey questions were analyzed using SPSS (IBM Corporation, Armonk, NY, USA) and reported as frequencies and percentage of participant responses for each question. Pre and post survey results from those questions were analyzed using a paired *t*-test in SPSS. Means were considered significantly different at *p* < 0.05.

## 3. Results

### 3.1. Loin Quality

A total of 96 chops from 12 loins (8 chops per loin; 2 chops per cooking method degree of doneness combination) were used for consumer sensory panels. Visual color scores averaged 3.58, visual marbling scores averaged 2.58, and subjective firmness scores averaged 2.67 for this population of loins (Table 1). Additionally, instrumental lightness (L*) averaged 48.89, redness (a*) averaged 5.85, and yellowness (b*) averaged 5.33 for this population of loins. The mean ultimate pH of the 12 loins was 5.70.

### 3.2. Demographics

Demographic summary data for 133 participants were provided (Table 2). White ethnic origin (73.48%) and ages between 36–55 years old (29.55%) were the majority of participants. Over 57% of all participants were over the age of 35 years old. Completed an advanced or graduate degree was the most common education level among participants (43.18%). Gender was slightly skewed toward female (54.55%) compared with males (45.45%).

### 3.3. Cooked Meat Color

There were no significant interactions (*p* ≥ 0.09) between cooking method and degree of doneness for cooked chop instrumental color (L*, a*, and b*) (Table 3). However, chops cooked using sous-vide were 0.34 a* units redder (*p* = 0.03) and 0.33 b* units less yellow (*p* = 0.01) than chops cooked on a grill. Additionally, chops cooked to 63 °C were 0.28 a* units redder (*p* = 0.01) and 0.31 b* units less yellow (*p* = 0.01) than chops cooked to 71 °C. The lack of interactions between cooking method and degree of doneness means the effects of cooking method and degree of doneness were additive. Therefore, chops cooked by sous-vide to 63 °C were the reddest (4.26) followed by sous-vide to 71 °C (4.00), grilled to 63 °C (3.94), and grilled to 71 °C (3.64) which allowed for direct testing of the original hypothesis.

### 3.4. Consumer Sensory Evaluation

There were multiple interactions between cooking method and degree of doneness for tenderness, juiciness, and overall acceptability. Therefore, percentage of participants with ratings in each category were expressed as interaction means (Table 4).

A greater (*p* < 0.001) percentage of participants rated a chop cooked sous-vide 63 °C (82.82%) as tender compared with all other cooking method and degree of doneness combinations. Additionally, a greater (*p* = 0.05) percentage of participants rated a chop cooked on the grill to 71 °C (22.03%) as not tender compared with chops cooked to 63 °C on either the grill or sous-vide. Chops cooked to 63 °C were rated as tender by a greater percentage (*p* < 0.0001) of participants than chops cooked to 71 °C. Additionally, a greater percentage (*p* < 0.0001) of chops cooked using sous-vide were rated as tender compared to chops cooked on the grill (Table 4).

A greater percentage (*p* < 0.001) of participants rated a chop cooked sous-vide 63 °C (55.83%) as juicy compared with all other cooking method and degree of doneness combinations. Additionally, chops cooked using sous-vide to 63 °C (1.47%) had the least percentage (*p* < 0.01) of participants rate them as not juicy compared with all other cooking method and degree of doneness combinations.

Furthermore, chops cooked on the grill had a greater (*p* = 0.01) percentage of participants rate chops as not juicy compared to chops cooked sous-vide. On the other hand, a greater percentage (*p* < 0.0001) of chops cooked to 63 °C were rated as juicy compared to chops cooked to 71 °C (Table 4).

Nearly 35% of participants rated chops cooked to 63 °C as flavorful compared to only 24.31% of participants rating chops cooked to 71 °C as flavorful. Cooking method did not influence participant sensory flavor (*p* ≥ 0.30), nor was there an interaction between cooking method and degree of doneness (*p* ≥ 0.16) for sensory flavor (Table 4).

A greater percentage (*p* = 0.01) of chops cooked using sous-vide to 63 °C (60.34%) were rated as acceptable compared with all other cooking method and degree of doneness combinations. Additionally, only 2.22% of chops cooked using sous-vide to 63 °C were rated not acceptable and a greater (*p* = 0.04) percentage of chops cooked using sous-vide regardless of degree of doneness were rated acceptable compared with chops cooked on the grill.

### 3.5. Survey Results

Question 1 asked participants to report on average how many times they ate pork each month (Table 5). The greatest percentage of participants (40.6%) responded more than once a week followed by 2–3 times a month (29.3%), once a week (23.3%), once a month (4.5%) and finally only 2.3% of participants reported they consumed pork less than once a month. Question 2 asked participants to report on average how many times they cooked pork each month. The greatest percentage of participants (30.1%) responded more than once a week followed by 2–3 times a month (28.6%), once a week (19.5%), once a month (12.8%) and finally only 9% of participants cook pork less than once a month.

Question 3 asked participants to select the methods they used to cook pork. Participants could choose multiple answers; therefore, the percentages are expressed as the number of responses divided by the total number of participants. The greatest percentage of participants (80.5%) reported stove top as their cooking method followed by oven (68.4%), grill (66.9%), slow cooker (54.9%), deep fryer (8.3%), air fryer (6.8%), sous-vide (6.0%), and finally only 3.0% of participants reported using other methods. Other cooking methods included smoker, microwave, and instapot. Question 4 asked participants to select ways they determine when pork is done and ready to consume (Table 5). Participants could choose multiple answers; therefore, the percentages are expressed as the number of responses divided by the total number of participants. The greatest percentage of participants (67.7%) reported looking at the color of the meat as their way to determine doneness followed by using a meat thermometer (50.4%), cooking for a specific amount of time (33.1%), cooking until juices run clear (30.8%), other (6.8%), and finally 3.0% of participants reported not checking to see if the pork is done. Other ways to check doneness included firmness of the meat and a combination of specific time and temperature.

Questions 5 asked participants to evaluate a set of photos and report which degree of doneness they preferred (Table 6). In the pre-survey, over 50% (53.4%) of participants choose medium pork chop as their preferred degree of doneness followed by medium well (19.5%), medium rare (16.5%), and 15.8% of participants preferred well done pork chops. Treating this as a continuous variable that ranged from 1 (rare) to 5 (well done), the mean preference for the pre-survey was 3.27 (Table 7). In the post-survey, only 43.6% of participants preferred medium pork followed by medium rare (37.6%), medium well (12%), well done (6.8%), and only 0.8% of participants preferred rare pork. Again, treating this as a continuous variable that ranged from 1 (rare) to 5 (well done), the mean preference for the post-survey was 2.84 (Table 7). Based on the results of a paired *t*-test analysis, a greater percentage (*p* < 0.01) of participants preferred less well-done pork after the consumer sensory panel than prior to the consumer sensory panel.

Question 6 asked participants why they chose the degree of doneness as the most preferential based on the provided photographs (Table 6). In the pre-survey, the greatest percentage of participants responded that they perceived their preferred degree of doneness to be the juiciest (34.6%) followed by safest to consume (33.8%), best texture (22.6%), best flavor (21.8%), and 5.3% of participants chose based on other reasons. After the consumer sensory evaluation, the greatest percentage of participants responded that they perceived their preferred degree of doneness to be the juiciest (45.1%) followed by best flavor (25.6%), best texture (24.1%), safest to consume (12.0%), and only 1.5% of participants choose that degree of doneness for other reasons. Question 7 asked participants to report at what temperature (in Fahrenheit) they believed pork was safe to consume (Table 7). In the pre-survey the average temperature was 154.43 °F (68.0 °C) and in the post-survey the average temperature was 144.84 °F (62.7 °C). A paired *t*-test found participants believed that pork was safe at a lower temperature in the post-survey compared to the pre-survey (*p* < 0.01).

## 4. Discussion

For the present study, participants were served chops cooked either using a broiling method (open hearth grill) or sous-vide cooking method. In a United States Food and Drug Administration (FDA) consumer survey, 52.3% of participants used a grill/barbecue when preparing pork chops [14]. Although the foodservice industry has been using sous-vide cooking method since the early 2000s, consumers are becoming more aware of the cooking method as an in-home option since approximately 2010 [7]. The sous-vide cooking method allows chefs to hold meat at a constant temperature over a long period of time without the meat warming above the desired temperature. Most commonly, chefs will cook using the sous-vide method then sear both sides of the meat to create a Maillard reaction [7,8]. Cooked meat flavor is a product of the Maillard reaction, which is a reaction between amino acids and reducing sugars [7]. However, the current study served sous-vide chops without any browning before or after the sous-vide cooking process.

The sous-vide method has been known to provide a tender and juicy product [1,7]. This held true with the current study as we saw a greater percentage of participants rating chops as tender and juicy for sous-vide cooking method compared to the grilling method. Sun et al. [15] reported that sous-vide cooking increased moisture retention relative to other cooking methods. Although, the initial hypothesis was that sous-vide would be rated less desirable compared to the broiling method due to the lack of browning, this was not the case. In fact, a greater percentage of participants rated chops cooked using sous-vide as acceptable compared to chops cooked using the grilling method. This increased acceptability rating is likely due to the increased ratings in tenderness and juiciness by participants for chops cooked using sous-vide compared to grilling.

In addition to cooking method, degree of doneness has the greatest impact on overall eating experience. In 2011, the USDA lowered the recommended final internal cooking temperature of whole muscle pork cuts from 71 °C to 63 °C [4]. In recent research, degree of doneness of pork loin chops has proven to have a superior impact on overall eating experience compared to quality traits such as pH, color, and marbling. Ultimate pH did not affect trained sensory tenderness scores when chops were cooked to 63 °C unless ultimate pH was above 5.95 [16]. Additionally, Wilson et al. [17] reported that color and marbling independently do not effect eating experience of trained sensory panelist when chops are cooked to 63 °C. Others reported a 1.75 increase in tenderness scores of trained sensory panels with decreasing internal cooking temperature [2]. Klehm et al. [18] reported a decrease in cooking loss by 1.64% when cooking chops to 63 °C compared to 71 °C. Rincker et al. [2] reported a 3.56 unit increase in juiciness scores of trained panelists with decreasing internal cooking temperature. Furthermore, a recent study reported that a greater percentage of consumers rated pork chops cooked to 63 °C as tender (81.25%), juicy (70.61%), flavorful (39.83%), and overall, more acceptable (61.82%) compared to pork chops cooked to 71 °C [3]. Chops used in this study were from loins that represented average pH, marbling, and color scores of chops found in U.S. retail cases [11]. In agreement with recent findings, the current study observed that participants preferred chops cooked to 63 °C compared to those cooked to 71 °C, regardless of cooking method as they were rated more tender and juicier. Improving palatability of pork chops in traits such as tenderness and juiciness are crucial to the pork industry in terms of protecting its market share from other protein sources. Based on data from this study cooking chops to 63 °C as opposed to 71 °C and cooking them using sous-vide appears to be a viable option.

In 1998, focus groups were conducted by the US Food Safety and Inspection Service (FSIS) to evaluate consumer behaviors. Consumers did not regularly use a meat thermometer and typically cooked pork until no red or pink was visible inside the meat [5]. The FDA food safety survey of 2016 indicated 33% of consumers do not own a meat thermometer and those that do own one only 38% use it on large pieces of meat and only 10% on hamburgers [14]. Results of the current study would suggest some progress has been made in the use of meat thermometers. Although, participants in this study most often reported that they determined when pork is done and ready to consume based on cooked color of the meat (67.7%), a larger percentage of participants than expected, about 50%, reported using a meat thermometer. For full adoption of sous-vide for at-home cooking it is likely perceptions of cooked pork will need to adjust.

According to the pre-survey of the present study, participants chose their preferred degree of doneness as medium. The greatest percentage for the reason why they chose that degree of doneness was because they thought it would be the juiciest (34.6%) followed by the safest to consume (33.8%). This was in line with the hypothesis for the present study where, when able to observe cooked color, consumers would prefer medium (71 °C) chops over medium-rare (63 °C) based on their pre-conceived opinions. By using white, as opposed to red light, the goal was to determine if these potential biased would negatively influence overall acceptability of pork when cooked to a lesser degree of doneness than some participants may be accustomed. Indeed, chops cooked to 63 °C were redder compared to chops cooked to 71 °C regardless of cooking method. In fact, chops cooked to 63 °C using sous-vide were the reddest of any treatment. The hypothesis was this increased redness would be perceived as being undercooked and thereby unsafe to consume and result in reduced acceptability scores.

However, even despite the redder color associated with cooking chops using sous-vide and cooking to a lesser degree of doneness, a greater percentage of participants rated chops cooked in this manner as being more acceptable than any other cooking method and degree of doneness combinations. Once participants were educated that 63 °C was safe to consume, post-survey results indicated that a greater percentage of participants chose medium-rare as their preferred degree of doneness. Additionally, the percentage of participants reasoning for choosing their degree of doneness preference decreased for safe to consume (12.0%) and increased for flavor (25.6%), texture (24.1%), and juicy (45.1%) from pre- to post-survey results. These results imply that consumers may still not be comfortable with the cooking recommendations provided by FSIS but become more comfortable and consider pork to provide a more satisfactory eating experience with pork cooked to 63 °C is obtained.

## 5. Conclusions

A lower final internal cooking temperature of pork chops increased the percentage of participants rating chops as tender, juicy, flavorful, and overall acceptable. Even when participants were given the opportunity to visually appraise the color of cooked chops, a greater percentage of participants preferred chops cooked to 63 °C compared to those cooked to 71 °C. Sous-vide chops were more tender, and a greater percentage rated acceptable compared to grilled chops. Further, the sous-vide 63 °C were the most tender, juicy, and acceptable compared with the other cooking method/degree of doneness combinations. Chops cooked sous-vide did not compromise acceptability or sensory traits of pork chops due to the lack of browning.

The survey results also indicated the consumer preferred a lower degree of doneness of pork after participating in the sensory panel and that they believed pork should be cooked to a lower temperature. Additionally, the participants were able to correctly identify the appropriate temperature to cook pork to for a more positive eating experience while still ensuring it is safe to consume.

Overall, participants preferred chops cooked to a lesser degree of doneness (63 °C) regardless of cooking method and preferred the sous-vide cooking method compared to grilling. The results of this study indicate the importance of degree of doneness and cooking method as well as educating consumers on the benefits of monitoring end point cooking temperature for a more positive eating experience.

## Figures and Tables

**Table 1:** Population summary statistics of fresh (not cooked) pork quality measurements of loins used to provide chops for sensory evaluation ^1^.

Variable	Number	Mean	Minimum	Maximum	SD	CV
Subjective evaluations ^2^						
Visual color	12	3.58	2.5	4.5	0.60	16.65
Visual marbling	12	2.58	1.5	3.5	0.60	23.10
Subjective firmness	12	2.67	2.0	3.0	0.49	18.46
Instrumental color ^3^						
Lightness, L*	12	48.89	43.90	52.54	2.98	6.10
Redness, a*	12	5.85	4.38	8.10	1.29	22.04
Yellowness, b*	12	5.33	2.80	7.01	1.23	23.12
Ultimate pH	12	5.70	5.50	6.08	0.21	3.61

^1^ Measurements were collected on chop 1 of each loin. ^2^ National Pork Producers Council (NPPC) color using the 1999 standards, half point scale where 1 = visually palest color and 6 = visually darkest color and where 1 = visually the least marbling and 6 = visually the most marbling. NPPC firmness using the 1991 standard where 1 = softest and 6 = firmest. ^3^ L* measures darkness to lightness (greater L* indicates a lighter color), a* measures redness (greater a* value indicates a redder color), and b* measures yellowness (greater b* value indicates a more yellow color).

**Table 2:** Demographic summary of participants (133 total) evaluating the effects of cooking method and degree of doneness of boneless pork chops.

Characteristic	Category	Percentage of Consumers
Age	18–25 years old	18
	26–35 years old	24
	36–55 years old	30
	56–75 years old	27
	76 years old or older	1
Ethnic origin	Asian/Pacific Islander	23
	Hispanic or Latino	2
	Other	2
	White	73
Gender	Female	55
	Male	45
Education level	Completed an advanced or graduate degree	43
	Some graduate school	17
	Completed an undergraduate degree	14
	Some college	22
	High school diploma	5

**Table 3:** Effects of cooking method and degree of doneness (DOD) on instrumental color of cooked pork chops.

	Cooking Method			Degree of Doneness			*p*-Value		
	Sous-Vide	Grill	SEM	63 °C	71 °C	SEM	Cooking Method	DOD	Cooking Method × DOD
Observations, n	24	24		24	24				
Lightness, L* ^1^	77.58	77.52	0.29	77.70	77.41	0.29	0.89	0.48	0.54
Redness, a* ^1^	4.13	3.79	0.09	4.10	3.82	0.09	0.03	0.01	0.88
Yellowness, b* ^1^	9.07	9.40	0.08	9.08	9.39	0.08	0.01	0.01	0.09
Cooked Temperature, (°C) ^2^	67.02	67.10	0.19	63.13	70.99	0.19	0.74	<0.01	0.13

^1^ L* measures darkness to lightness (greater L* indicates a lighter color), a* measures redness (greater a* value indicates a redder color), and b* measures yellowness (greater b* value indicates a more yellow color). ^2^ Final internal temperature recorded on each chop after cooking.

**Table 4:** Effects of cooking method and degree of doneness (DOD) on consumer sensory traits of pork chops ^1,2^.

	Sous-Vide		Grill			*p*-Value		
	63 °C	71 °C	63 °C	71 °C	SEM	Cooking Method	DOD	Cooking Method × DOD
Tenderness								
Not tender	1.4 ^c^	16.0 ^ab^	10.0 ^b^	22.0 ^a^	4.26	<0.01	<0.01	0.05
Neutral	15.9 ^b^	50.0 ^a^	51.5 ^a^	54.6 ^a^	4.35	<0.01	<0.01	<0.01
Tender	82.8 ^a^	33.1 ^bc^	37.7 ^b^	22.4 ^c^	4.68	<0.01	<0.01	<0.01
Juiciness								
Not juicy	1.5 ^c^	34.8 ^a^	14.9 ^b^	27.9 ^a^	4.70	0.01	<0.01	<0.01
Neutral	42.5	53.9	50.9	51.7	4.55	0.49	0.17	0.23
Juicy	55.8 ^a^	10.9 ^d^	33.5 ^b^	19.8 ^c^	4.90	0.62	<0.01	<0.01
Flavor								
Not flavorful	16.0	26.1	16.8	18.3	4.27	0.38	0.12	0.26
Neutral	45.2	51.3	51.3	54.3	4.58	0.30	0.30	0.73
Flavorful	38.2	22.1	31.2	26.7	4.81	0.88	0.01	0.16
Acceptability								
Not acceptable	2.2 ^c^	22.8 ^a^	12.0 ^b^	21.2 ^a^	4.14	0.02	<0.01	0.01
Neutral	37.2	50.1	48.6	49.3	4.50	0.22	0.12	0.16
Acceptable	60.3 ^a^	26.4 ^c^	38.6 ^b^	28.6 ^bc^	4.87	0.04	<0.01	0.01

^a–d^ Least squares means within a row among main effects and interaction means lacking a common superscript differ (*p* < 0.05). ^1^ Values reported are a percentage of responses for each of the interaction means. ^2^ Consumers used a 9-point Likert-type score system where scores 1 through 3 were considered not tender, not juicy, not flavorful, or unacceptable. Scores 4 through 6 were considered neutral for tenderness, juiciness, flavor, and overall acceptability. Scores 7 through 9 were considered tender, juicy, flavorful, and acceptable.

**Table 5:** Frequency and percentage of participants response to pre-survey questions (133 total).

	Frequency	Percentage
How many times do you eat pork each month?		
More than once a week	54	40.6
2–3 times a month	39	29.3
Once a week	31	23.3
Once a month	6	4.5
Less than once a month	3	2.3
How many times do you cook pork each month?		
More than once a week	40	30.1
2–3 times a month	38	28.6
Once a week	26	19.5
Once a month	17	12.8
Less than once a month	12	9.0
Which of the following ways do you use to cook pork?		
Stove top	107	80.5
Oven	91	68.4
Grill	89	66.9
Slow Cooker	73	54.9
Deep Fryer	11	8.3
Air Fryer	9	6.8
Sous-vide	8	6.0
Other	4	3.0
How do you determine when the pork is done and ready to consume?		
Look at the color of the meat	90	67.7
Use a meat thermometer	67	50.4
Cook for a specific amount of time	44	33.1
Cook until juice runs clear	41	30.8
Other	9	6.8
I do not check to see if it is done	4	3.0

**Table 6:** Frequency and percentage of participants response to pre-survey and post-survey questions.

	Pre-Survey		Post-Survey	
	Frequency	Percentage	Frequency	Percentage
After looking at the photos, which of the following degree of doneness do you prefer?	133 total		133 total	
Medium	71	53.4	58	43.6
Medium Well	26	19.5	16	12.0
Medium Rare	22	16.5	50	37.6
Well Done	21	15.8	9	6.8
Rare	0	0.0	1	0.8
What is the main reason for why you chose the degree of doneness photo?	133 total		133 total	
Juiciest	46	34.6	60	45.1
Safest to consume	45	33.8	16	12.0
Best texture	30	22.6	32	24.1
Best flavor	29	21.8	34	25.6
Other	7	5.3	2	1.5

**Table 7:** Comparison of pre-survey and post-survey questions regarding degree of doneness preference and safe temperature to consume pork ^1^.

	Pre-Survey		Post-Survey	
Survey Questions	Mean	SD	Mean	SD
Which of the following would you prefer to eat? ^1^	3.27	0.9	2.84	0.87
At what temperature (°F) do you think pork is safe to consume?	154.43	13.8	144.84	6.3

^1^ Degree of doneness definitions were given numerical numbers: 1 = Rare; 2 = Medium Rare; 3 = Medium; 4 = Medium Well; 5 = Well Done.

## Data Availability

Reasonable requests for datasets generated from the current experiment are available from the corresponding author.
